# Use of C-reactive protein for the early prediction of anastomotic leak after esophagectomy: Systematic review and Bayesian meta-analysis

**DOI:** 10.1371/journal.pone.0209272

**Published:** 2018-12-17

**Authors:** Alberto Aiolfi, Emanuele Asti, Emanuele Rausa, Giulia Bonavina, Gianluca Bonitta, Luigi Bonavina

**Affiliations:** Department of Biomedical Sciences for Health, Division of General Surgery, University of Milan, IRCCS Policlinico San Donato, Milan, Italy; Baylor College of Medicine, UNITED STATES

## Abstract

**Background:**

Early suspicion, diagnosis, and timely treatment of anastomotic leak after esophagectomy is essential. Retrospective studies have investigated the role of C-reactive protein (CRP) as early marker of anastomotic leakage. The aim of this systematic review and meta-analysis was to evaluate the predictive value of CRP after esophageal resection.

**Methods:**

A literature search was conducted to identify all reports including serial postoperative CRP measurements to predict anastomotic leakage after elective open or minimally invasive esophagectomy. Fully Bayesian meta-analysis was carried out using random-effects model for pooling diagnostic accuracy measures along with CRP cut-off values at different postoperative day.

**Results:**

Five studies published between 2012 and 2018 met the inclusion criteria. Overall, 850 patients were included. Ivor-Lewis esophagectomy was the most common surgical procedure (72.3%) and half of the patients had squamous-cell carcinoma (50.4%). The estimated pooled prevalence of anastomotic leak was 11% (95% CI = 8–14%). The serum CRP level on POD3 and POD5 had comparable diagnostic accuracy with a pooled area under the curve of 0.80 (95% CIs 0.77–0.92) and 0.83 (95% CIs 0.61–0.96), respectively. The derived pooled CRP cut-off values were 17.6 mg/dl on POD 3 and 13.2 mg/dl on POD 5; the negative likelihood ratio were 0.35 (95% CIs 0.096–0.62) and 0.195 (95% CIs 0.04–0.52).

**Conclusion:**

After esophagectomy, a CRP value lower than 17.6 mg/dl on POD3 and 13.2 mg/dl on POD5 combined with reassuring clinical and radiological signs may be useful to rule-out leakage. In the context of ERAS protocols, this may help to avoid contrast radiological studies, anticipate oral feeding, accelerate hospital discharge, and reduce costs.

## Introduction

Esophageal resection, the therapeutic gold-standard in esophageal carcinoma, carries high morbidity and mortality rates that have remained unchanged in the minimally invasive surgery era [1]. Pneumonia and anastomotic leakage still represent the major postoperative complications, despite significant heterogeneity in definition [2–5]. Early suspicion of anastomotic leak is desirable to exclude patients from enhanced recovery pathways, thereby delaying oral feeding and improving the prognosis of sub-clinical leaks [6].

Inflammatory biomarkers like C-reactive protein (CRP), procalcitonin, and white blood cell count have been proposed for early diagnosis of surgical and infectious complications after major surgery [7–12]. A previous systematic review and meta-analysis has shown that CRP is a useful negative predictive test to rule out anastomotic leak in elective colorectal surgery [13]. However, despite the introduction of complex risk models, the clinical utility of biomarkers to predict anastomotic leaks after esophagectomy has never been consistently demonstrated, and no previous meta-analyses on this topic have been performed yet [14–15].

The aim of this systematic review and Bayesian meta-analysis was to investigate the role of CRP as predictive biomarker of anastomotic leak in patients undergoing elective esophagectomy for carcinoma.

## Materials and methods

We conducted this study according to the Preferred Reporting Items for Systematic Reviews and Meta-analyses (PRISMA) statement [16]. An extensive literature search, until May 31^st^ 2018, was conducted by two independent authors (AA, ER) to identify the English-written published series on the predictive value of CRP level for anastomotic leakage in patients who underwent elective esophageal resection for cancer. Pubmed, MEDLINE, Embase, and Cochrane databases were consulted matching the terms “esophagectomy” OR “esophageal resection” AND “C-reactive protein” OR “CRP”. The reference lists of all relevant articles were searched manually to identify further relevant studies.

**Fig 1:**
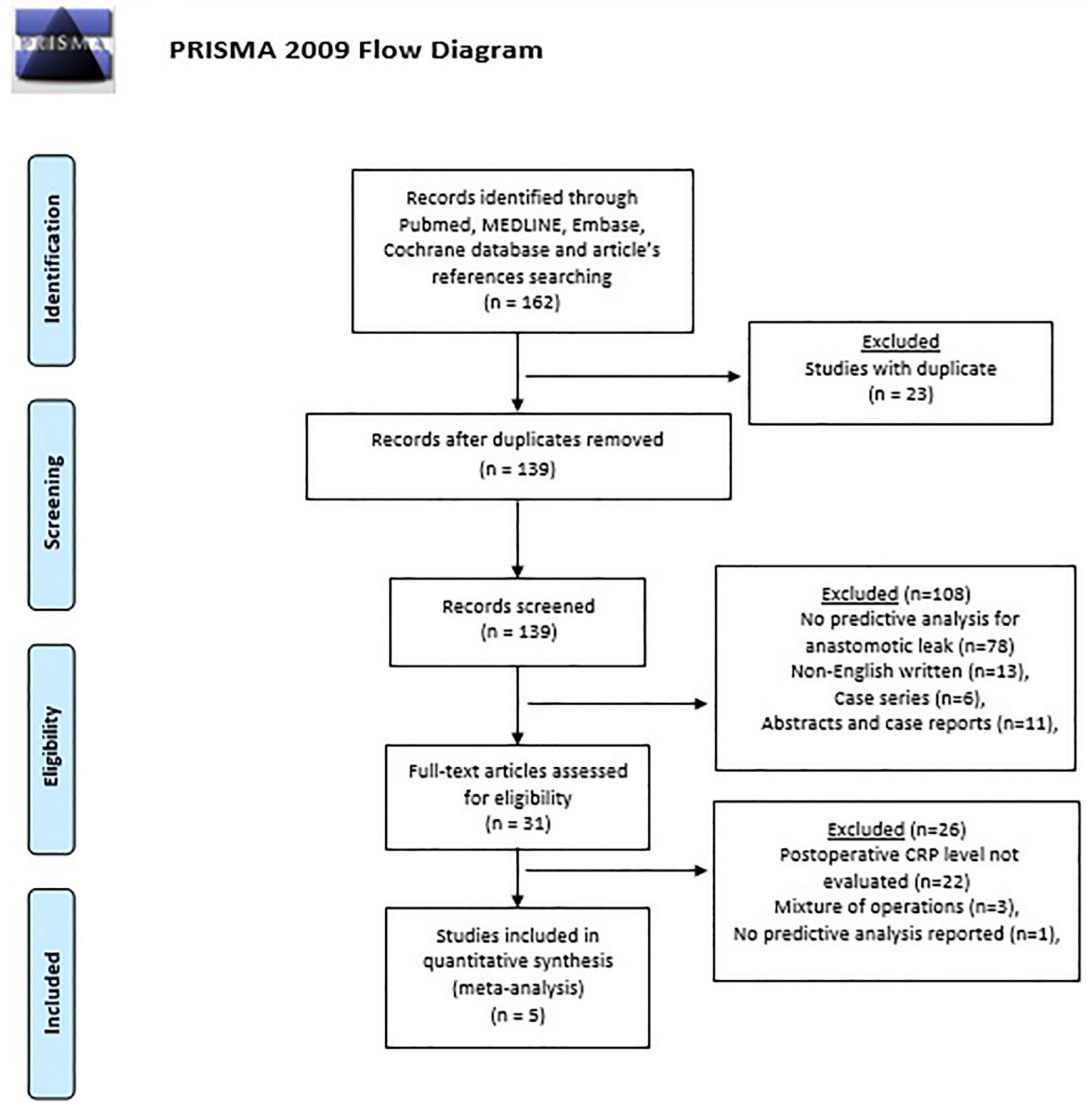
The Preferred Reporting Items for Systematic Reviews and Meta-Analyses (PRISMA) diagram.

Abstracts, case reports, case series, and non-English written articles were excluded. Relevant studies not allowing a predictive analysis for anastomotic leak were excluded (Fig 1). Two authors (AA, ER) independently extracted data from eligible studies. Data extracted included study characteristics (first author name, year, journal of publication), number of patients, time frame, demographic and preoperative clinical characteristics, surgical approach, and postoperative outcomes. The outcome of interest was anastomotic leakage, which was counted per event and defined as reported in the included studies. Measures of diagnostic accuracy, including area under the receiver operating characteristic curve (AUC), sensitivity, specificity, positive predictive value (PPV) and negative predictive value (NPV), were recorded to enable a diagnostic meta-analysis to be performed. To obtain a summary graph of postoperative CRP levels, CRP data reported in the text, graphs or figures of the included studies were used and/or digitalized to obtain the median or mean CRP value on each POD. Corresponding authors were contacted to obtain the necessary data when they were not available from the article. Disagreements between authors were resolved by consensus; if no agreement could be reached, a third senior author (LB) made the decision.

Three investigators independently evaluated the methodological quality of the papers using the Quality Assessment of Diagnostic Accuracy Studies (QUADAS-2) tool [17]. This assessed the risk of bias and concerns about applicability by evaluating four key domains: patient selection, index test, reference standard, and flow of patients through the study and timing of tests.

### Statistical analysis

Bivariate meta-analysis was conducted using a fully Bayesian approach via integrated nested Laplace approximations (INLA). Compared to traditional meta-analyses, the Bayesian approach takes into account all sources of variation and reflects these variations in the pooled result [18–19]. Furthermore, the Bayesian approach can provide more accurate estimates for small samples [20]. Chu and Cole bivariate generalized linear mixed effects with exact binomial likelihood model was used to summarize the results of several diagnostic studies by modelling sensitivity and specificity jointly (binomial-normal model) [21]. We assume that both sensitivity and specificity was modelled with the same logit link function. Normal prior with zero mean and 100 variance is used for the fixed effects. Variance components of random effect were modelled using penalized complexity priors choosing the parameters believing that the sensitivities or specificities lie in the interval [0.5, 0.95] with probability 0.95, according to Wakefield [22–23]. The binomial-normal model was also used to calculate the hierarchical summary receiver operating characteristic (HSROC) model according to Rutter and Gatsonis [24]. Uniform distribution on [-1,1] was the choice for a vague prior of the random effects correlation parameter. Pooled likelihood ratios and pooled diagnostics odds ratio (DOR) where computed sampling from approximated posterior distribution. The 95% bias-corrected and accelerated (BCa) bootstrap confidence interval for cut-off. The pooled prevalence of anastomotic leak was calculated as described elsewhere [25]. Standard error for postoperative CRP levels was estimated with the GetData Graph Digitizer software by two independent authors (AA, ER) [26]. The pooled mean CRP dosages in different postoperative days were estimated using Bayesian normal likelihood model with inverse gamma non informative prior for variability. Credible intervals (CIs) were computed. Statistical significance is set when 95% CIs involved. All analyses and figures were carried out using R software package version 3.4.3 [27].

## Results

### Systematic review

Five studies published between 2012 and 2018 met the inclusion criteria. Overall, 850 patients were included (range 45–258). All reports were observational, cohort studies. The definition of esophageal anastomotic leak used in the individual studies is reported in Table 1. Demographic, clinical, and operative variables of the patient sample are shown in Table 2. Patients’ age ranged from 37 to 85 years, and the majority (82.5%) were males. The ASA score was reported in five studies and the BMI in four studies. Esophagectomy was performed via an open or minimally invasive approach; the most common procedure was the Ivor-Lewis (72.3%) followed by the McKeown esophagectomy (14.6%). Squamous-cell carcinoma was the most common histological type (50.4%) followed by adenocarcinoma (46.9%). Tumor histology was not reported in one study. Only one study reported the results stratified according to the use of neoadjuvant therapy; the other studies did not differentiate the patients and results were reported as aggregated. The overall anastomotic leak rate was 11.5%.

**Table 1:** Definition of anastomotic leak.

Reference	Definition/diagnosis of anastomotic leak
Noble et al. [28]	Leak sufficient to cause symptoms and confirmed by radiology (contrastenhanced multi-detector CT scan with on-table oral contrast or water-soluble contrast studies), endoscopy or surgical exploration
Hoeboer et al. [9]	Esophagoenteric leak confirmed by endoscopy or esophageal contrast videography that requires local treatment, surgical treatment, or removal of conduit.
Gordon et al. [29]	Extravasation of oral contrast material seen on cross-sectional imaging or an anastomotic defect visualized intraoperatively on return to theatre. Endoscopy was not used to diagnose AL.
Park et al. [11]	Disruption of the anastomosis that leads to outflow of the intraluminal content, which is obvious leaks, as well as leaks without the presence of any clinical symptoms but with only occult leaks detected with esophagography followed by chest CT.
Asti et al. [6]	Anastomotic leakage was suspected by the presence of clinical signs and confirmed by extravasation of oral contrast at gastrographin swallow study and/or CT scan, and/or visualization of anastomotic defect at upper gastrointestinal endoscopy.

**Table 2:** Summary of the studies included in the meta-analysis. nr: not reported. SCC: Squamous cell carcinoma. ADK Adenocarcinoma. IVL: Ivor-Lewis esophagectomy. LTA: left thoracotomic approach.

Reference	Study design	No. patients	Mean age	M/F	Histology (n)	Neoadjuvant therapy (n)	Surgical approach (n)	Anastomotic leak (n)
Noble et al, 2012 [28]	Retrospective	258	67 (37–85)	202, 56	nr	156	IVL (112), McKeown (51), LTA (52), Transhiatal (43)	26
Hoeboer et al, 2015 [9]	Prospective	45	62.5 ± 15	39, 6	SCC (11), ADK (31), Other (3)	40	Transhiatal (16), Transthoracic (29)	10
Gordon et al, 2016 [29]	Retrospective	103	60 ± 15	79, 24	SCC (9), ADK (90), Other (4)	nr	IVL (103)	10
Park et al, 2017 [11]	Retrospective (non-NT)	156	63.9 ± 9	184, 17	SCC (201)	0	IVL (170), McKeown (31)	15
	Retrospective (NT)	45				45		8
Asti et al, 2018 [6]	Retrospective	243	61.7 ± 16	197, 46	SCC (77), ADK (157), Other (9)	96	IVL (201), McKeown (42)	29

**Fig 2:**
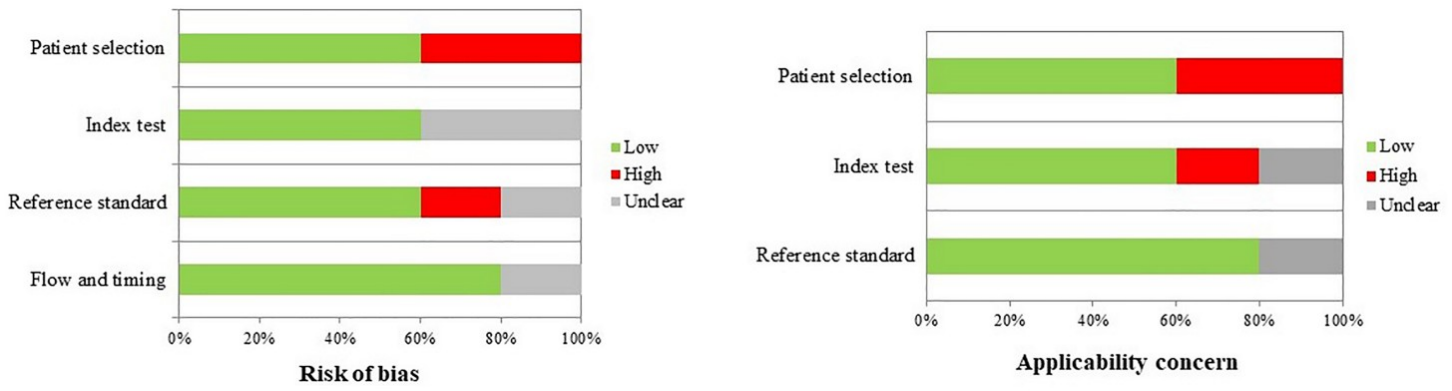
Quality assessment (QUADAS-2). Proportion of studies with low, high, or unclear risk of bias, %.

**Fig 3:**
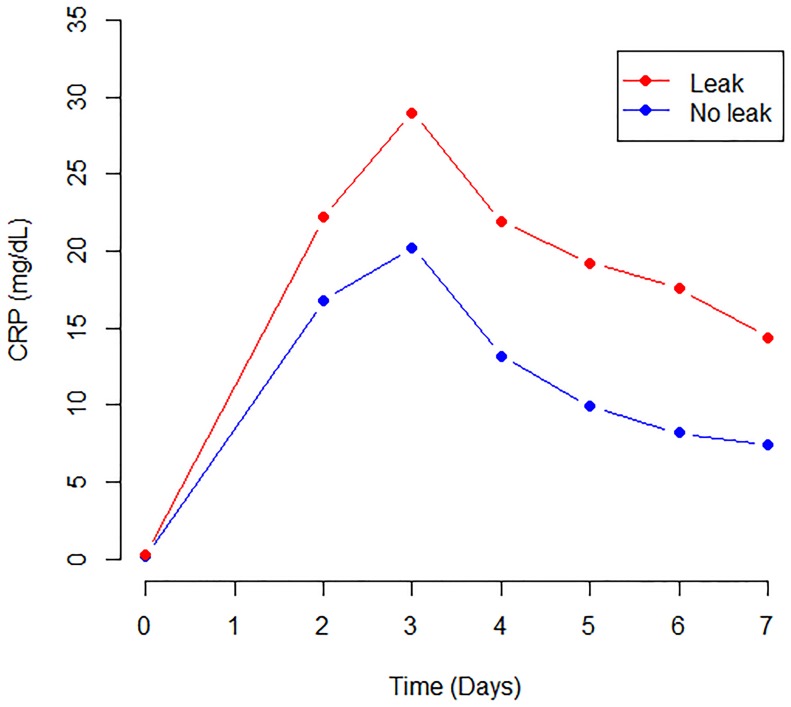
Pooled post-operative CRP levels in the two patients groups (*p*<0.05).

The results of quality assessment using the QUADAS-2 tool are shown in Fig 2. Overall, the applicability of included studies was good. The included studies reported measuring CRP in the postoperative period according to different institutional protocols. Patients were stratified on the presence of anastomotic leak (AL group) or no complications (NC group). Five studies reported CRP levels on POD2, 5 studies on POD3, 4 studies on POD4, 5 studies on POD5, 4 studies on POD6 and 5 studies on POD7. Cut-off CRP values were reported in 5 studies on POD3, 2 studies on POD4, and 3 studies on POD5. The pooled postoperative CRP dosages in the two groups are showed in Fig 3 (*p*<0.05).

### Meta-analysis

**Fig 4:**
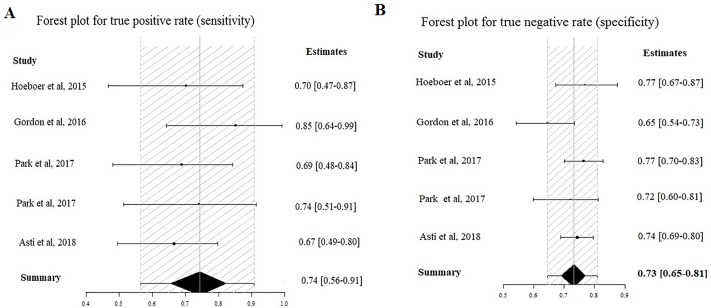
Forrest plot for POD3: Estimated pooled sensibility (A) and specificity (B).

**Fig 5:**
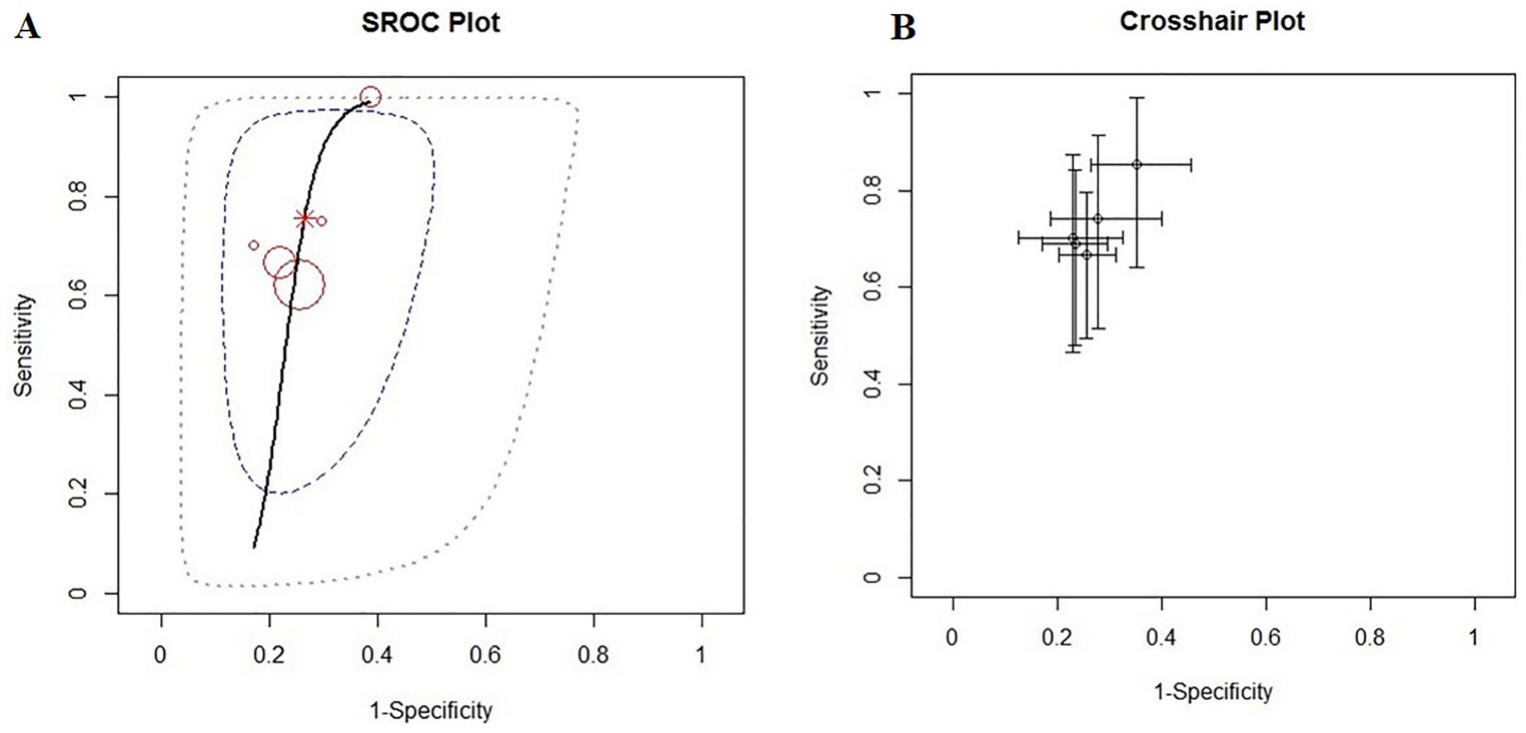
Summary ROC curve (A), and cross air plot (B) for POD3.

In addition to a systematic review, we performed a study-level fully Bayesian meta-analysis. Considering the random effect bivariate model, the estimated pooled POD3 CRP cut-off, resulting from 4 studies and 592 patients, is 17.6 mg/dl (95% CI 14.9–20 mg/dl). The estimated pooled sensibility is 0.74 (95% CIs 0.56–0.91) (Fig 4a) and the pooled specificity is 0.73 (95% CIs 0.65–0.81) (Fig 4b). The estimated pooled AUC is 0.80 (95% CIs 0.77–0.92). The pooled positive LR is 2.78 (95% CIs 1.87–4.09) and the pooled negative LR is 0.35 (95% CIs 0.096–0.62). The diagnostic OR is 8.0 (95% CIs 3.20–32.70). The summary ROC curve and the summary estimates of sensitivity and specificity are reported in Fig 5a. The cross air plot showed a non-typical shoulder arm appearance, indicating the absence of threshold effect due to difference in cut-off values (Fig 5b). The calculated correlation between sensitivity and specificity was 0.24 suggesting no threshold effect.

**Fig 6:**
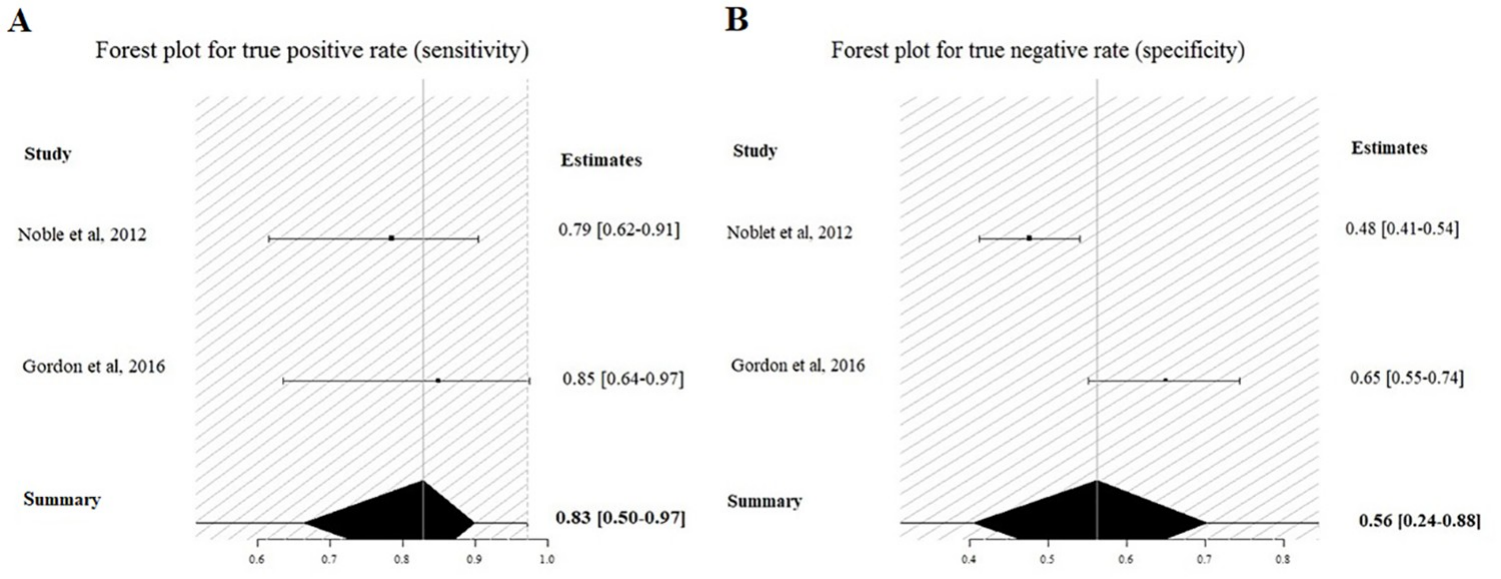
Forrest plot for POD4: Estimated pooled sensibility (A) and specificity (B).

**Fig 7:**
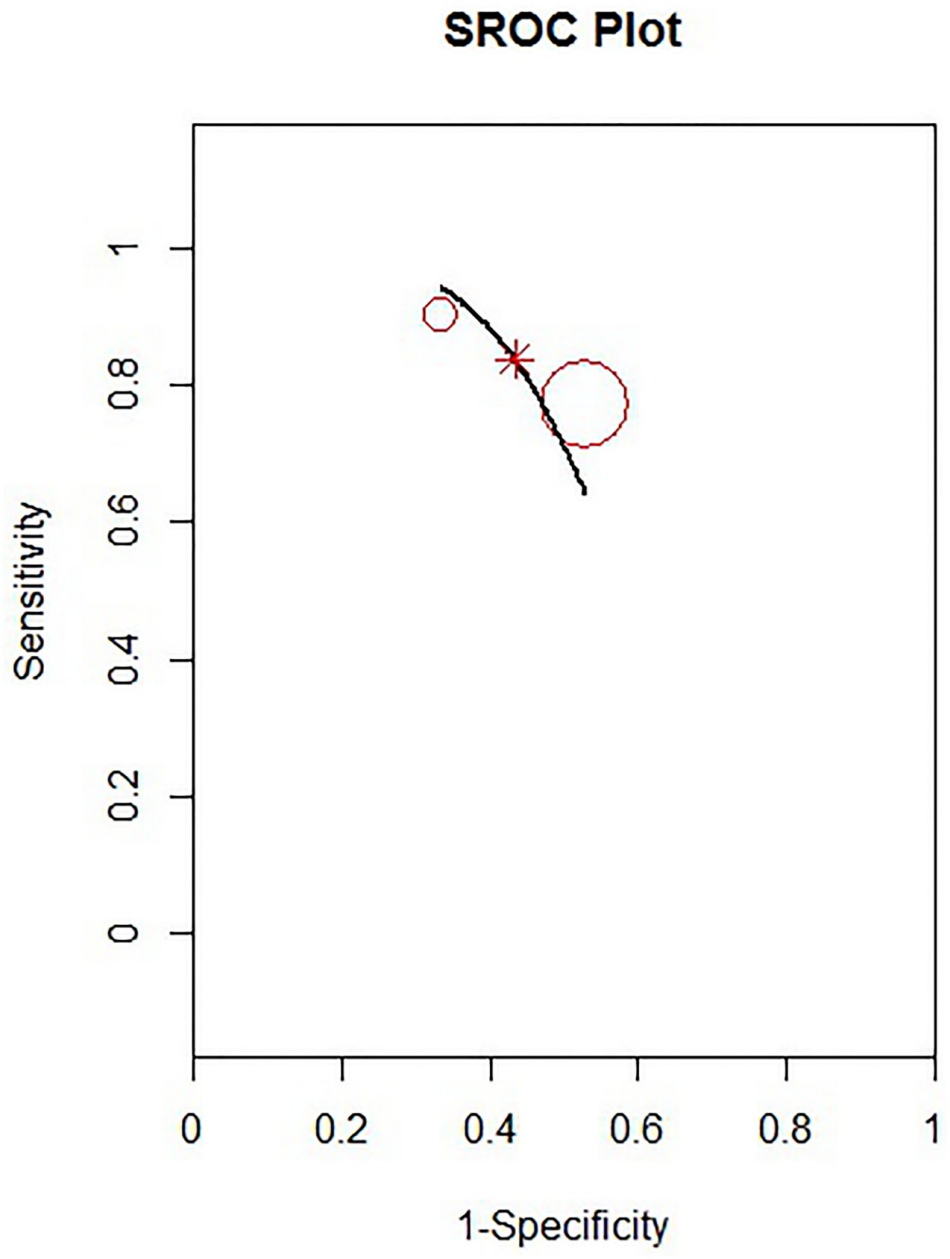
Summary ROC curve for POD4.

The estimated pooled POD4 CRP cut-off, resulting from 2 studies with 361 patients, is 17.7 mg/dl (95% CI 17.3–17.65 mg/dl). The pooled sensibility is 0.83 (95% CIs 0.50–0.97) (Fig 6a) and the pooled specificity is 0.56 (95% CIs 0.24–0.85) (Fig 6b). The estimated pooled AUC is 0.82 (95% CIs 0.77–0.98). The summary ROC curve and the summary estimates of sensitivity and specificity are reported in Fig 7. The pooled positive LR is 1.88 (95% CIs 0.81–7.0) and the pooled negative LR is 0.30 (95% CIs 0.03–1.49). The diagnostic OR is 6.53 (95% CIs 0.48–128.64). The calculated correlation between sensitivity and specificity was 0.075 suggesting no threshold effect.

**Fig 8:**
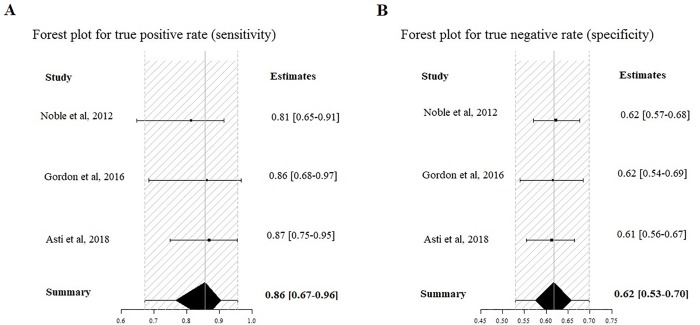
Forrest Plot for POD5: Estimated pooled sensibility (A) and specificity (B).

**Fig 9:**
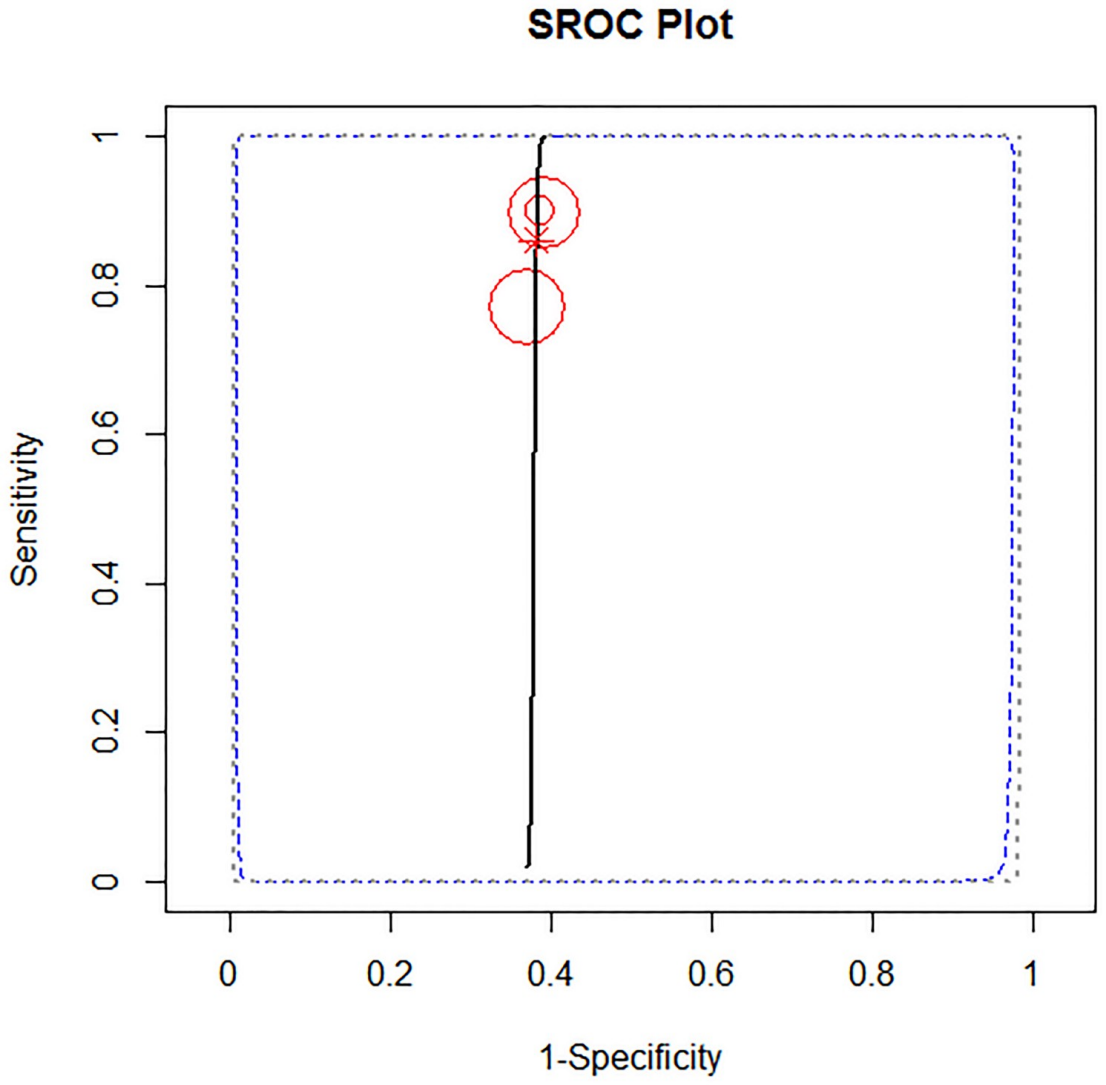
Summary ROC curve for POD5.

The estimated pooled POD5 CRP cut-off, resulting from 3 studies with 604 patients, is 13.2 mg/dl (95% CI 8.3–16.7 mg/dl). The estimated pooled sensitivity is 0.86 (95% CIs 0.67–0.96) (Fig 8a) and the pooled specificity is 0.62 (95% CIs 0.53–0.70) (Fig 8b). The estimated pooled AUC is 0.83 (95% CIs 0.61–0.96). The summary ROC curve and the summary estimates of sensitivity and specificity are reported in Fig 9. The pooled positive LR is 2.22 (95% CIs 1.48–3.00) and the pooled negative LR is 0.195 (95% CIs 0.04–0.52). The diagnostic OR is 9.66 (95% CIs 2.51–46.99). The calculated correlation between sensitivity and specificity was 0.022, suggesting no threshold effect.

**Fig 10:**
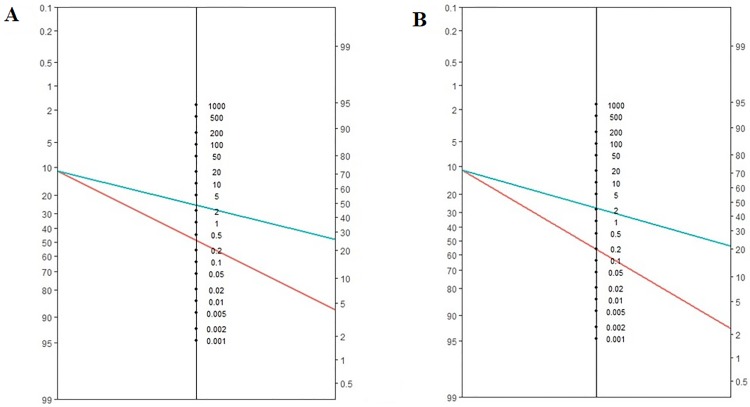
The Fagans’ nomograms for POD3 (A) and POD5 (B).

The estimated pooled prevalence of anastomotic leak resulting from 5 studies, which include a total of 850 patients, is 11% (95% CI = 8–14%). The Fagans’ nomograms for POD3 and POD5 are showed in Fig 10.

## Discussion

This systematic review and meta-analysis shows that serum CRP concentration measured on POD3, POD4, and POD5 after esophagectomy may be useful test to rule out anastomotic leakage. After surgery, many patients who do not develop anastomotic leak may reveal a severe systemic inflammatory response with increased CRP levels related to the severity of surgical trauma, blood loss, and duration of operation [30–32]. Therefore, CRP is mostly valuable as a negative test, and a low CRP level on POD3 and POD5 may help to predict patients who are unlikely to develop an anastomotic leak.

The pooled incidence of anastomotic leak was 11% (95% CI = 8–14%). The peak value of CRP occurred on POD3 and was significantly higher in the AL compared to the NC group. The pooled CRP cut-off value on POD3, POD4, and POD5 were 17.6 mg/dl, 17.7 mg/dl, and 13.2 mg/dl respectively. The diagnostic accuracy is supported by the pooled AUC ROC curves. The negative predictive value, which is indeed useful to measure the accuracy of a predictive test, appears less consistent when applied to an individual patient. Conversely, LRs are more precise in estimating the diagnostic probability of a single test, thus providing an individual risk assessment. Therefore, in clinical practice, positive LR is relevant to confirm (rule-in), while negative LR is relevant to exclude disease/complication (rule-out) [33–34]. The pooled positive LRs indicate a weak evidence to diagnose anastomotic leak on POD 3 (LR+ 2.78), POD4 (LR+ 1.88), and POD 5 (LR+ 2.22). On the other hand, the LR- showed moderate evidence to rule-out leakage on POD 5 (LR- 0.195). This means that for a low-risk patient, with a pretest probability of 11% and a negative test on POD5 (CRP <13.2 mg/dl), the probability of having an anastomotic leak is almost 2% (Fig 10). Notably, the lower limit of CIs of LR- for CRP on POD3, POD 4, and POD5 were 0.096, 0.03 and 0.06, respectively. This suggests that, in the absence of clinical and/or radiological suspicion, CRP may provide solid evidence to rule out leakage.

Despite an overall significant progress in the management of esophageal cancer, anastomotic leak potentially remains a fatal consequence of esophagectomy. Early detection and treatment of this complication is critical to optimize perioperative care, minimize surgical complications, and expediting recovery. It has been shown that the application of Enhanced Recovery after Surgery (ERAS) programs for esophagectomy is associated with favorable overall morbidity and mortality, and reduced hospital length of stay [35–40]. However, the methodological quality of these studies is limited [41–42]. Identifying a clinically relevant CRP cut-off may be a helpful adjunct to the fast-track pathways by providing an early alert for leakage, selecting patients for diagnostic studies, and tailoring the therapeutic interventions. Integration of a CRP-based algorithm in clinical practice may lead to reduction of clinical burden and costs associated with anastomotic leak.

The main result of the present study was a significant association between postoperative CRP levels and anastomotic leak. However, given the relevant sensibility, specificity and the AUC combined with significant LR- on POD3 and POD5, postoperative CRP values may be useful to exclude (rule-out) rather than diagnose (rule-in) anastomotic leak after elective esophagectomy. Therefore, in the context of a fast-track recovery protocol, CRP could be used for early diet advancement and safe discharge home, respectively [6]. On the other hand, early diagnosis of anastomotic leakage by radiology and/or endoscopy is critical to provide immediate treatment by means of antibiotic therapy, placement of naso-jejunal feeding tube, stenting with or without percutaneous drainage, or endoVAC therapy, possibly reducing the rate of surgical revision [43–44].

Owing to differences in patient population, study design and methodology, this meta-analysis is limited by the heterogeneity of the included studies. Four of the five studies were retrospective and one had a prospective design. In addition, the various surgical approaches and techniques, the different definitions of anastomotic leak, and the effect of neoadjuvant treatment may contribute to inter-study heterogeneity [45]. However, postoperative CRP measurements were performed in all patients, thus reducing the possibility of diagnostic accuracy overestimation. Results for POD 4 were limited due to the fact that only two studies were included. Finally, it should be considered that longitudinal studies with repeated measurements taken over time are more reliable in establishing causality [46]. To the best of our knowledge, this is the first meta-analysis that assessed the prognostic value of CRP to rule-out anastomotic leak after esophageal resection. Although meta-analysis is not a widely approved method for summarizing predictive data, the cut-off values recommended in the present study can be used to interpret postoperative CRP measurements and may be integrated in ERAS protocols. Further prospective, high-quality studies are needed to validate the results of this meta-analysis.

## Conclusions

Increased postoperative levels of CRP may be associated with anastomotic leakage after esophagectomy. A CRP value lower than cut-off values of 17.6 mg/dl on POD3 and 13.2 mg/dl on POD5 may be useful to rule-out leakage. In the context of ERAS protocols, this may help to avoid contrast radiological studies, anticipate oral feeding, accelerate hospital discharge, and reduce costs.

## Supporting information

S1 Checklist A checklist displaying which PRISMA items are described on what page of the manuscript. (DOC)

## References

[1] Schmidt HM, Gisbertz SS, Moons J, Rouvelas I, Kauppi J, Brown A, et al Defining benchmarks for transthoracic esophagectomy. A multicenter analysis of total minimally invasive esophagectomy in low risk patients. *Ann Surg* 2017; 11;266(5):814–821. 10.1097/SLA.0000000000002445 28796646 10.1097/SLA.0000000000002445

[2] Low DE, Alderson D, Cecconello I, Chang AC, Darling GE, DʼJourno XB, et al International consensus on standardization of data collection for complications associated with esophagectomy. *Ann Surg* 2015;262:286–294. 10.1097/SLA.0000000000001098 25607756 10.1097/SLA.0000000000001098

[3] Kassis ES, Kosinski AS, Ross P Jr, Koppes KE, Donahue JM, Daniel VC. Predictors of anastomotic leak after esophagectomy: an analysis of the society of thoracic surgeons general thoracic database. *Ann Thorac Surg* 2013;96:1919–26. 10.1016/j.athoracsur.2013.07.119 24075499 10.1016/j.athoracsur.2013.07.119

[4] Bonavina L, Scolari F, Aiolfi A, Bonitta G, Sironi A, Saino G, et al Early outcome of thoracoscopic and hybrid esophagectomy: propensity-matched comparative analysis. *Surgery* 2016;159:1073–1081. 10.1016/j.surg.2015.08.019 26422764 10.1016/j.surg.2015.08.019

[5] Biere SS, van Berge Henegouwen MI, Bonavina L, Rosman C, Roig Garcia J, Gisbertz SS, et al Predictive factors for post-operative respiratory infections after esophagectomy for esophageal cancer: outcome of randomized trial. *J Thorac Dis* 2017;9:S861–S867. doi: 10.21037/jtd.2017.06.61 28815084 10.21037/jtd.2017.06.61 PMC5538980

[6] Asti E, Bonitta G, Melloni M, Tornese S, Milito P, Sironi A, et al Utility of C-reactive protein as predictive biomarker of anastomotic leak after minimally invasive esophagectomy. *Langenbecks Arch Surg*. 2018 3;403(2):235–244. 10.1007/s00423-018-1663-4 29516256 10.1007/s00423-018-1663-4

[7] Straatman J, Harmsen AM, Cuesta MA, Berkhof J, Jansma EP, van der Peet DL. Predictive value of C-Reactive Protein for major complications after major abdominal surgery: a systematic review and pooled-analysis. *PLoS One* 2015;10:e0132995 10.1371/journal.pone.0132995 26177542 10.1371/journal.pone.0132995 PMC4503561

[8] Adamina M, Steffen T, Tarantino I, Beutner U, Schmied BM, Warschkow R. Meta-analysis of the predictive value of C-reactive protein for infectious complications in abdominal surgery. *Br J Surg* 2015;102:590–598. 10.1002/bjs.9756 25776855 10.1002/bjs.9756

[9] Hoeboer SH, Groeneveld AB, Engels N, van Genderen M, Wijnhoven BP, van Bommel J. Rising C-Reactive Protein and Procalcitonin levels precede early complications after esophagectomy. *J Gastrointest Surg* 2015;19:613–624. 10.1007/s11605-015-2745-z 25663633 10.1007/s11605-015-2745-z PMC4361731

[10] Miki Y, Toyokawa T, Kubo N, Tamura T, Sakurai K, Tanaka H, et al C-Reactive Protein indicates early stage of postoperative infectious complications in patients following minimally invasive esophagectomy. *World J Surg* 2017;41:796–803. 10.1007/s00268-016-3803-8 27878351 10.1007/s00268-016-3803-8

[11] Park JK, Kim JJ, Moon SW. C-reactive protein for the early prediction of anastomotic leak after esophagectomy in both neo-adjuvant and non-neoadjuvant therapy case: a propensity score matching analysis. *J Thorac Dis* 2017;9(10):3963–702. doi: 10.21037/jtd.2017.08.125 29268376 10.21037/jtd.2017.08.125 PMC5723863

[12] Warschkow R, Tarantino I, Ukegjini K, Beutner U, Müller SA, Schmied BM, et al Diagnostic study and meta-analysis of C-reactive protein as a predictor of postoperative inflammatory complications after gastroesophageal cancer surgery. *Langenbecks Arch Surg* 2012; 397: 727–736. 10.1007/s00423-012-0944-6 22398436 10.1007/s00423-012-0944-6

[13] Singh PP, Zeng IS, Srinivasa S, Lemanu DP, Connolly AB, Hill AG. Systematic review and meta-analysis of use of serum C-reactive protein levels to predict anastomotic leak after colorectal surgery. *Br J Surg*. 2014 3;101(4):339–46. 10.1002/bjs.9354 24311257 10.1002/bjs.9354

[14] Findlay JM, Tustian E, Millo J, Klucniks A, Sgromo B, Marshall RE, et al The effect of formalizing enhanced recovery after esophagectomy with a protocol. *Dis Esophagus*. 2015;28(6):567–73. 10.1111/dote.12234 24835109 10.1111/dote.12234

[15] Paireder M, Jomrich G, Asari R, Kristo I, Gleiss A, Preusser M, et al External validation of the NUn score for predicting anastomotic leakage after oesophageal resection. *Sci Rep*. 2017;7(1):9725 10.1038/s41598-017-10084-9 28852063 10.1038/s41598-017-10084-9 PMC5575338

[16] Moher D, Liberati A, Tetzlaff J, Altman DG; PRISMA Group. Preferred reporting items for systematic reviews and meta-analyses: the PRISMA statement. *PLoS Med*. 2009;21;6(7):e1000097 10.1371/journal.pmed.1000097 19621072 10.1371/journal.pmed.1000097 PMC2707599

[17] Whiting PF, Rutjes AW, Westwood ME, Mallett S, Deeks JJ, Reitsma JB, et al QUADAS-2: a revised tool for the quality assessment of diagnostic accuracy studies. *Ann Intern Med* 2011; 155: 529–536. 10.7326/0003-4819-155-8-201110180-00009 22007046 10.7326/0003-4819-155-8-201110180-00009

[18] Mila AL, Ngugi HK. A Bayesian approach to meta-analysis of plant pathology studies. *Phytopathology*. 2011;101:42–51. 10.1094/PHYTO-03-10-0070 20822433 10.1094/PHYTO-03-10-0070

[19] Sutton AJ, Abrams KR. Bayesian methods in meta-analysis and evidence synthesis. Stat. Methods Med. Res. 2001;10:277–303. 10.1177/096228020101000404 11491414 10.1177/096228020101000404

[20] Higgins JP, Thompson SG, Spiegelhalter DJ. A re-evaluation of random-effects meta-analysis. *J R Stat Soc Ser A Stat Soc* 2009;172:137–159. 10.1111/j.1467-985X.2008.00552.x 19381330 10.1111/j.1467-985X.2008.00552.x PMC2667312

[21] Chu H, Cole SR. Bivariate meta-analysis of sensitivity and specificity with sparse data: A generalized linear mixed model approach. J Clin Epidemiol. 2006 12;59(12):1331–1332. 10.1016/j.jclinepi.2006.06.011 17098577 10.1016/j.jclinepi.2006.06.011

[22] Simpson DP, Martins TG, Riebler A, et al. H. Sørbye. Penalising model component complexity: A principled, practical approach to constructing priors. ArXiv e-prints, 2014

[23] Wakefield J. Disease mapping and spatial regression with count data. *Biostatistics*, 8(2): 158–183, 2007 10.1093/biostatistics/kxl008 16809429 10.1093/biostatistics/kxl008

[24] Rutter CM, Gatsonis CA. A hierarchical regression approach to meta-analysis of diagnostic test accuracy evaluations. *Statistics in Medicine*, 20(19):2865–2884, 2001 11568945 10.1002/sim.942

[25] Asti E, Sozzi M, Bonitta G, Bernardi D, Bonavina L. Esophagectomy in patients with liver cirrhosis: a systematic review and Bayesian meta-analysis. *J Visc Surg*. 2018 4 10 [Epub ahead of print]. 10.1016/j.jviscsurg.2018.03.014 29653854 10.1016/j.jviscsurg.2018.03.014

[26] http://getdata-graph-digitizer.com Accessed February 20th 2018

[27] R Core Team. R: a language and environment for statistical computing. R Foundation for Statistical Computing [cited 2016 Mar 14]. www.r-project.org Accessed May 31, 2018

[28] Noble F, Curtis N, Harris S, Kelly JJ, Bailey IS, Byrne JP, et al Risk assessment using a novel score to predict anastomotic leak and major complications after oesophageal resection. *J Gastrointest Surg*. 2012 6;16(6):1083–1095. 10.1007/s11605-012-1867-9 22419007 10.1007/s11605-012-1867-9

[29] Gordon AC, Cross AJ, Foo EW, Roberts RH. C-reactive protein is a useful negative predictor of anastomotic leak in oesophago-gastric resection. ANZ J Surg. 2018 3;88(3):223–227. 10.1111/ans.13681 27457697 10.1111/ans.13681

[30] Pittet D, Rangel-Frausto S, Li N, Tarara D, Costigan M, Rempe L, et al Systemic inflammatory response syndrome, sepsis, severe sepsis and septic shock: incidence, morbidities and outcomes in surgical ICU patients. *Intensive Care Med* 1995; 21: 302–309. 7650252 10.1007/BF01705408

[31] Haga Y, Beppu T, Doi K, Nozawa F, Mugita N, Ikei S, et al Systemic inflammatory response syndrome and organ dysfunction following gastrointestinal surgery. *Crit Care Med* 1997; 25: 1994–2000. 9403749 10.1097/00003246-199712000-00016

[32] Pepys MB, Hirschfield GM. C-reactive protein: a critical update. *J Clin Invest* 2003; 111: 1805–1812. 10.1172/JCI18921 12813013 10.1172/JCI18921 PMC161431

[33] Akobeng AK. Understanding diagnostic tests 2: likelihood ratios, pre- and post-test probabilities and their use in clinical practice. *Acta Paediatr* 2007; 96: 487–491. 10.1111/j.1651-2227.2006.00179.x 17306009 10.1111/j.1651-2227.2006.00179.x

[34] Halkin A, Reichman J, Schwaber M, Paltiel O, Brezis M. Likelihood ratios: getting diagnostic testing into perspective. *QJM* 1998; 91: 247–258. 9666947 10.1093/qjmed/91.4.247

[35] Cerfolio RJ, Bryant AS, Bass CS, Alexander JR, Bartolucci AA. Fast tracking after Ivor Lewis esophagogastrectomy. *Chest*. 2004 10;126(4):1187–94. 10.1378/chest.126.4.1187 15486381 10.1378/chest.126.4.1187

[36] Low DE, Kunz S, Schembre D, Otero H, Malpass T, Hsi A, et al Esophagectomy—it's not just about mortality anymore: standardized perioperative clinical pathways improve outcomes in patients with esophageal cancer. *J Gastrointest Surg*. 2007 11;11(11):1395–1402. 10.1007/s11605-007-0265-1 17763917 10.1007/s11605-007-0265-1

[37] Jiang K, Cheng L, Wang JJ, Li JS, Nie J. Fast track clinical pathway implications in esophagogastrectomy. *World J Gastroenterol*. 2009 1 28;15(4):496–501. 10.3748/wjg.15.496 19152457 10.3748/wjg.15.496 PMC2653358

[38] Munitiz V, Martinez-de-Haro LF, Ortiz A, Ruiz-de-Angulo D, Pastor P, Parrilla P. Effectiveness of a written clinical pathway for enhanced recovery after transthoracic (Ivor Lewis) oesophagectomy. *Br J Surg*. 2010 5;97(5):714–718. 10.1002/bjs.6942 20187171 10.1002/bjs.6942

[39] Li C, Ferri LE, Mulder DS, Ncuti A, Neville A, Lee L, et al An enhanced recovery pathway decreases duration of stay after esophagectomy. *Surgery*. 2012 10;152(4):606–614. 10.1016/j.surg.2012.07.021 22943844 10.1016/j.surg.2012.07.021

[40] Cao S, Zhao G, Cui J, Dong Q, Qi S, Xin Y, et al Fast-track rehabilitation program and conventional care after esophagectomy: a retrospective controlled cohort study. *Support Care Cancer*. 2013 3;21(3):707–714. 10.1007/s00520-012-1570-0 22933129 10.1007/s00520-012-1570-0

[41] Findlay JM, Gillies RS, Millo J, Sgromo B, Marshall RE, Maynard ND. Enhanced recovery for esophagectomy: a systematic review and evidence-based guidelines. *Ann Surg*. 2014 3;259(3):413–431. 10.1097/SLA.0000000000000349 24253135 10.1097/SLA.0000000000000349

[42] Parise P, Ferrari C, Cossu A, Puccetti F, Elmore U, De Pascale S, et al Enhanced Recovery After Surgery (ERAS) Pathway in Esophagectomy: Is a Reasonable Prediction of Hospital Stay Possible? *Ann Surg*. 2018 4 18 [Epub ahead of print]. 10.1097/SLA.0000000000002775 29672400 10.1097/SLA.0000000000002775

[43] Bonavina L, Asti E, Sironi A, Bernardi D, Aiolfi A. Hybrid and total minimally invasive esophagectomy: how I do it. J Thorac Dis. 2017 7;9(Suppl 8):S761–S772. doi: 10.21037/jtd.2017.06.55 28815072 10.21037/jtd.2017.06.55 PMC5538984

[44] Rausa E, Asti E, Aiolfi A, Bianco F, Bonitta G, Bonavina L. Comparison of endoscopic vacuum therapy versus endoscopic stenting for esophageal leaks: systematic review and meta-analysis. *Dis Esophagus*. 2018 6 25 [Epub ahead of print] 10.1093/dote/doy060 29939229 10.1093/dote/doy060

[45] Yamashita K, Watanabe M, Mine S, Toihata T, Fukudome I, Okamura A, et al Minimally invasive esophagectomy attenuates the postoperative inflammatory response and improves survival compared with open esophagectomy in patients with esophageal cancer: a propensity score matched analysis. *Surg Endosc*. 2018 4 11 10.1007/s00464-018-6187-z 29644466 10.1007/s00464-018-6187-z

[46] Frees EW. Longitudinal and Panel Data: Analysis and Applications in the Social Sciences. 2004 New York: Cambridge University Press.

